# Antibacterial Performance of Protonated Polyaniline-Integrated Polyester Fabrics

**DOI:** 10.3390/polym14132617

**Published:** 2022-06-28

**Authors:** Muhammad Faiz Aizamddin, Mohd Muzamir Mahat, Zaidah Zainal Ariffin, Mohd Azizi Nawawi, Nur Aimi Jani, Nur Asyura Nor Amdan, Kishor Kumar Sadasivuni

**Affiliations:** 1 School of Physics and Material Studies, Faculty of Applied Sciences, Universiti Teknologi MARA, Shah Alam 40450, Malaysia; faizaizamddin@gmail.com (M.F.A.); nuraimi_jani@uitm.edu.my (N.A.J.); 2 School of Biology, Faculty of Applied Sciences, Universiti Teknologi MARA, Shah Alam 40450, Malaysia; drzaidah@uitm.edu.my; 3 School of Chemistry and Environmental Studies, Faculty of Applied Sciences, Universiti Teknologi MARA, Shah Alam 40450, Malaysia; azizi_nawawi@uitm.edu.my; 4 Bacteriology Unit, Infectious Disease Research Centre, Institute for Medical Research, National Institutes of Health, Setia Alam, Shah Alam 40170, Malaysia; asyura@moh.gov.my; 5 Center for Advanced Materials, Qatar University, Doha P.O. Box 2713, Qatar; kishorkumars@qu.edu.qa

**Keywords:** antibacterial fabric, conductive fabric, antioxidant activity, polyaniline, polyester fabric

## Abstract

During the last few years, there has been an increase in public awareness of antimicrobial fabrics, as well as an increase in commercial opportunities for their use in pharmaceutical and medical settings. The present study reports on the optimized fabrication of protonated polyaniline (PANI)-integrated polyester (PES) fabric. *Para*-toluene sulfonic acid (*p*TSA) was used to protonate the PANI fabric and thus grant it antibacterial performance. The results of a 1,1-diphenyl-2-picrylhydrazyl (DPPH) scavenging assay showed high antioxidant activity of protonated PANI fabric at a scavenging efficiency of 84.83%. Moreover, the findings revealed remarkably sensitive antibacterial performance of PANI-integrated fabric against the following Gram-positive bacteria: methicillin-resistant *Staphylococcus aureus* (MRSA), *S. epidermidis,* and *S. aureus*; and also against the following Gram-negative bacteria: *P. aeruginosa*, *E. coli*, and *S. typhi*. Attenuated total reflectance–Fourier transform infrared (ATR–FTIR) spectroscopy and energy dispersive X–ray fluorescence (EDXRF) were used to determine the changes in the structural and elemental compositions of PANI fabric upon treatment with bacterial strains. Electrochemical impedance spectroscopy (EIS) revealed that the electrical conductivity value of protonated PANI fabric decreased by one (1) order of magnitude against *P. aeruginosa* and *S. aureus*, from 3.35 ± 7.81 × 10^−3^ S cm^−1^ to 6.11 ± 7.81 × 10^−4^ S cm^−1^ and 4.63 ± 7.81 × 10^−4^ S cm^−1^, respectively. Scanning electron microscopy (SEM) analysis showed the disruption of bacterial membranes and their structures when exposed to protonated PANI fabric; meanwhile, thermogravimetric analysis (TGA) demonstrated that the fabric retained its thermal stability characteristics. These findings open up potential for the use of antimicrobial fabrics in the pharmaceutical and medical sectors.

## 1. Introduction

Modified fabrics, such as those of cotton, nylon, and polyester (PES), have recently been introduced into the cosmetic, pharmaceutical, electronic, and medicinal industries [1,2]. These fabrics have been integrated with designed antimicrobial compounds for therapeutic, controlled release, protection, sensing, and monitoring applications. Antibacterial functional fabrics are receiving more attention in research and development in the search to reduce health risks associated with infectious bacteria and epidemic outbreaks throughout the world. There are goods in hospitals and households that use fabrics, such as medical apparel and surgical attire. This includes medical clothing, surgical wear, mattress covers, curtains, bedding, and wipes. Consequently, infusing antibacterial substances into the fabrics is considered essential in some applications that must limit the spread of bacteria in contaminated environments [3,4,5].

Electrostatic interactions between cations and bacteria’s anionic charge at their membrane surfaces, or other interactions between cations and bacteria’s RNA/DNA proteins, are commonly cited as the mechanism for antibacterial activity [6,7,8,9,10]. Moreover, Zafari et al. [11] reported that the surface hydrophobicity of bacteria also plays an important role in bacteria’s inactivation and stress condition when exposed to antibacterial agents. In order to combat bacterial growth, the antibacterial fabric usually contains positively charged materials in the form of either small molecules, nanoparticles, or metal ions such as copper, silver, zinc, etc. [9,12,13]. However, Sánchez-López et al. [14] asserted that there are risks to humans and the environment that come with this approach. Instead, incorporating conducting polymers (CPs) via chemical or electrochemical methods on the fabric surface provides a safe and preferable method of producing a fabric with antibacterial properties.

The polymer’s oxidized backbone, such as polyaniline (PANI), is stabilized by the alternation of charged ions, which donate or take electrons. The protonated state of CPs is determined by the extent of the doping agents used; for example, phytic acid (PA), *para*-toluene sulfonic acid (*p*TSA), hydrochloric acid (HCl), and phosphoric acid [15,16,17,18]. Moreover, the production of hydroxyl radicals between PANI and its environment leads to the formation of hydrogen peroxide, which can destroy cells [19]. Moreover, the electrostatic adherence between the PANI molecules and bacteria plays a very important role in the antibacterial reaction of a PANI fabric. Despite acting as a charge carrier, these fabrics were reported to grant antibacterial properties by introducing dopant ions into their structure. Robertson et al. [20] claimed that novel functionalised PANI has an active reaction against *E. coli* strains. Omar et al. [21] did a study recently that found that phytic acid-incorporated PANI fabrics could have a lot of antibacterial power against clinical pathogens such as *K. pneumonia, S. aureus* and *E. coli*. Jarach et al. [22] in their study demonstrated a 100% elimination of *S. aureus* and *S. epidermidis* strains by PANI-integrated fabric. From the aforementioned reports, PANI fabric could become a superior prospect as a future antibacterial agent.

Here, we present the optimised fabrication of PANI-integrated PES fabric that utilizes *p*TSA as a dopant for its antibacterial performance. While there are major studies which are investigating how protonated PANI fabric conveys an antibacterial effect and previous research has only focused on limited types of bacteria [23,24,25]. Collectively, there is an important point to emphasize: each Gram-positive and Gram-negative bacteria type may have different antibacterial activities against PANI fabric. Therefore, this study covered the antibacterial assessment against the following Gram-positive bacteria: methicillin-resistant *Staphylococcus aureus* (MRSA), *Staphylococcus epidermidis* (*S. epidermidis*)*, Staphylococcus aureus* (*S. aureus*); and against the following Gram-negative bacteria: *Pseudomonas aeruginosa* (*P. aeruginosa*), *Escherichia coli* (*E. coli*), and *Salmonella typhi* (*S. typhi*). A 1,1-diphenyl-2-picrylhydrazyl (DPPH) radical-scavenging test was performed to quantify the antioxidant activity of the fabric. The tentative mechanisms and effects that contribute to the antibacterial properties are also discussed.

Attenuated total reflectance–Fourier transform infrared (ATR–FTIR) spectroscopy and energy dispersive X–ray fluorescence (EDXRF) were employed to determine the changes in the structural and elemental compositions of PANI fabric upon treatment with bacterial strains. Meanwhile, electrochemical impedance spectroscopy (EIS) was performed to characterize the fabric’s electrical properties. The morphological changes of the bacterial surface membrane were analysed with a scanning electron microscope (SEM), while thermogravimetric analysis (TGA) was conducted in order to reveal the extent to which the virgin fabric’s thermal stability was maintained. These findings report promising antibacterial performance of the fabric as a result of its inherent dopants, such as *p*TSA, and the conjugated backbone of PANI-integrated fabric.

## 2. Materials and Methods

### 2.1. Materials

Bare polyester (PES) fabric (50 × 60 cm^2^) was purchased from a local retailer, Kamdar Sdn. Bhd. (Klang, Malaysia). Polyaniline (PANI) solution and *para*-toluene sulfonic acid (*p*TSA) were sourced from Sigma Aldrich (St. Louis, MO, USA).

### 2.2. Preparation of Protonated PANI Fabric

PANI-integrated PES fabric was fabricated through an immersion method as reported in our previous studies [17]. Briefly, a bare piece of PES fabric was cut to specified (5 × 5 cm^2^) dimensions and then submerged in a 10-millilitre green solution of protonated PANI (emeraldine salt). Formerly, PANI was protonated by chemically doped *p*TSA, in which the optimized value was 0.9 weight percentage (wt.%) concentration [16]. The immersion process was carried out for 30 min after which the fabric was dried at room temperature for 24 h in dark conditions. For deprotonated PANI (emeraldine base)-integrated fabric, no addition of *p*TSA was employed. 

### 2.3. DPPH Radical-Scavenging Assay

A 1,1-diphenyl-2-picrylhydrazyl (DPPH) radical-scavenging activity was used to quantify the antioxidant activity of each fabric. Briefly, 10 mg of the fabric was added to 1.6 mL of 0.1 mM DPPH-methanol solution. The blank was set at 517 nm, and the absorbance of the fabric was recorded after a 30-minute incubation period at a temperature of 25 °C in dark conditions. A lower absorbance indicates stronger DPPH radical-scavenging activity in the reaction mixture. The test was carried out in triplicate. The scavenging activity of the fabric in percent (%) was calculated using the following formula [26]:

Scavenging activity (%) = (A_blank_ − A_fabric_/A_blank_) × 100

where A_blank_ is the absorbance rate of the bare PES fabric (control), and A_fabric_ is the absorbance of the tested fabric. The spectrum was analysed using an ultra-violet spectrophotometer (model: Secomam Prim Light, Aqualabo Group, Shanghai, China).

### 2.4. Statistical Analysis

Results of the DPPH radical-scavenging assay are presented as means ± standard deviations (SD). An analysis of ordinary one-way ANOVA was carried out in order to compare significant relationships between three (3) groups with one (1) parameter. ANOVAs were corrected using Tukey’s hypothesis test in comparing bare PES fabric (control group) with undoped PANI fabric and *p*TSA-doped PANI fabric. A difference was considered significant if *p* < 0.0001. The statistical analysis was conducted using GraphPad Prism version 9.0 (GraphPad Software, San Diego, CA, USA).

### 2.5. Kirby–Bauer Disk Diffusion

Three isolates of Gram-positive bacteria (methicillin-resistant *Staphylococcus aureus* (MRSA) (BAA 2094), *Staphylococcus epidermidis* (*S. epidermidis*) (ATCC12228), and *Staphylococcus aureus* (*S. aureus*) (ATCC25923)) were demonstrated. Meanwhile, Gram-negative bacteria, namely *Escherichia coli* (*E. coli*) (ATCC1129), *Pseudomonas aeruginosa* (*P. aeruginosa*) (ATCC10145), and *Salmonella typhi* (*S. typhi*) (ATCC14028), were used to perform the antibacterial performance test of PANI-integrated fabrics. The bacteria isolates (4.0 × 10^−7^ cfu/mL) were inoculated in MH broth and incubated at 37 °C for 24 h under shaking conditions (250 rpm). The bacterial suspension’s turbidity was adjusted to that of a McFarland standard of 0.5 by adding PBS (1×). The MH agar was lawned with each bacterial isolate using a sterile cotton swab. The specimens (6.05 ± 0.08 mm) were placed onto the agar with sterilized tweezers, including the control antibiotic discs for each strain (Table 1), bare PES fabric, deprotonated PANI fabric, and protonated PANI fabric. The plates with lawned bacteria and specimens were incubated at 37 °C for 18 h. The appearance and size of the inhibition zone around the specimens were observed and measured. Measurements were performed in triplicate.

### 2.6. Characterisation of Protonated PANI-Integrated Fabrics

#### 2.6.1. Attenuated Total Reflectance–Fourier Transform Infrared (ATR–FTIR) Analysis in Collaboration with Energy Dispersive X–ray Fluorescence (EDXRF) Spectroscopy 

The FTIR spectra were studied with a Perkin Elmer Spectrum 100 ATR–FTIR. The analysis was performed at room temperature with ambient humidity. The spectra were recorded from 450 cm^−1^ to 4000 cm^−1^ at a frequency of 20 kHz, using a resolution of 16 cm^−1^. Each spectrum was recorded using a mirrored diamond surface under spectroscopic circumstances. The spectra were analysed with BIO–RAD WIN–IR PRO software (version 10, Perkin Elmer, Ltd., London, UK) in order to identify the functional group components within the PANI-integrated fabric. Meanwhile, EDXRF (model: Epsilon 3XLE–EDXRF spectrometer, Malvern Panalytical Ltd., Cambridge, UK) analysis was performed to measure the elemental composition of the PANI fabric. K X–rays were used to irradiate the fabric three (3) times, with the average value recorded; the voltage used was 50 kV. Nitrogen (N) and sulfur (S) were specifically selected as elements of interest.

#### 2.6.2. Electrochemical Impedance Spectroscopy (EIS)

The electrical conductivity of PANI fabric was measured with EIS (model: HIOKI 3532–50 LCR–HI Tester, HIOKI E. E. Corporation, Nagano, Japan). The analysis was performed at room temperature using a frequency range from 100 Hz to 1000 kHz. PANI-integrated fabric (5 × 5 cm^2^) was clamped between two copper electrodes, each with a 1-centimetre diameter. The thickness of the fabric was measured beforehand with a digital thickness gauge. An average of three measurements was recorded. Conductivity measurements were derived from the following expression [9,27,28]:

Ơ = L/(R_b_ × A)

where L is the thickness of the conductive fabric measured with a digital thickness gauge; R_b_ is the bulk resistance of the fabric extracted from the Nyquist data; and A is the area of the electrodes.

#### 2.6.3. Scanning Electron Microscopy (SEM) Analysis

The surface morphology of PANI-integrated fabric was observed with an SEM (model: SNE–4500M Plus Tabletop SEM, SEC Co., Ltd., Suwon-Si, Korea). Before this observation, a ~10-nanometre gold (Au) film was sputtered onto the samples in order to improve the image’s resolution. The fabric was cut with a razor blade before the images were captured, using magnifications ranging from 3000× to 10,000×.

#### 2.6.4. Thermogravimetric Analysis (TGA)

A thermogravimetric analyzer (TGA; SETARAM model, KEP Technologies, Sophia Antipolis, France) was used to elucidate the thermal stability profiles of the PANI-integrated fabric pieces. The fabrics were heated at a rate of 20 °C/min, from 27 °C to 300 °C. The results of the thermal reaction were amalgamated into a graph that shows how much weight was lost with temperature or time.

## 3. Results and Discussion

### 3.1. Fabrication of PANI Fabrics

**Figure 1:**
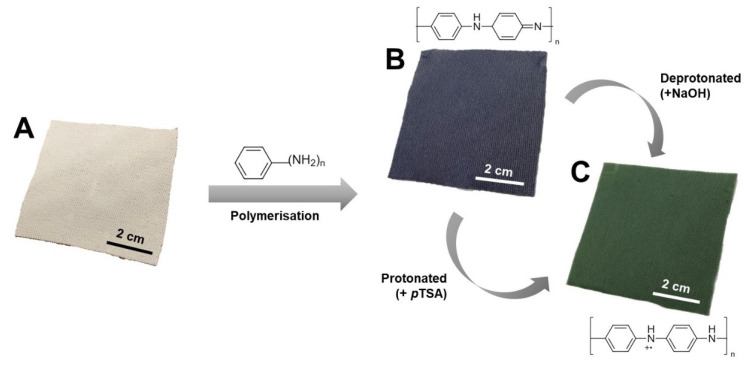
Close-up image of (**A**) bare PES fabric, (**B**) deprotonated, and (**C**) protonated PANI-integrated fabric.

The white appearance on the bare PES fabric in Figure 1A was constant. During the immersion process in undoped PANI, the fabric turned a blue color (Figure 1B). This blue-colored PANI portrays the deprotonated state (emeraldine base), which is considered a non-conductive state [29,30,31]. Meanwhile, as shown in Figure 1C, immersion in PANI doped with *p*TSA produced green fabric. The fabric color change to green indicates a successful doping process resulting from the modulation of acid that generates charge carriers into the PANI backbone. According to a past study by Mahat et al. [18], the green shading indicates the emerald salt state, which could alter the electrical conductivity of PANI and possibly grant antibacterial performance. Nevertheless, this method of doping is reversible, depending on the environment. PANI’s characteristics such as electrical performance may be lost if de-doping (the removal of dopant) is performed with alkaline conditions (e.g., sodium hydroxide (NaOH) or physiological conditions with a pH > 7.4).

### 3.2. Antioxidant Activity of PANI Fabrics

**Figure 2:**
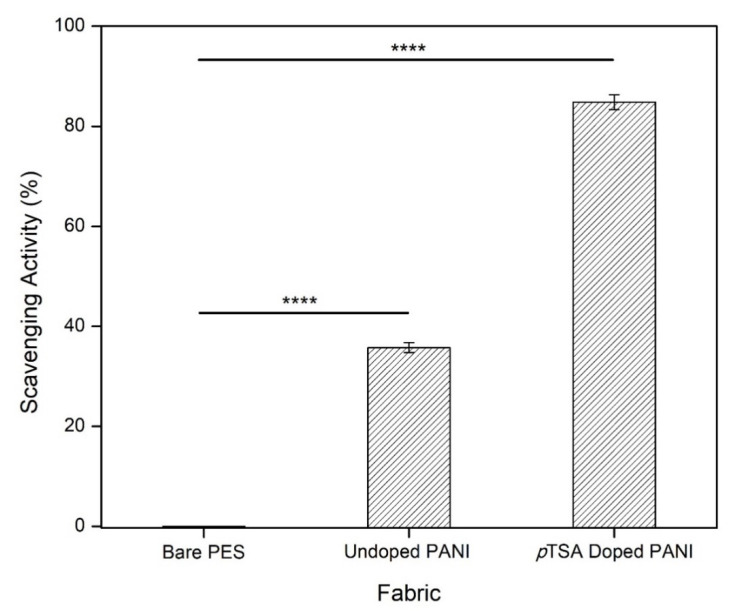
The antioxidant activity of bare PES fabric (control), undoped PANI fabric, and *p*TSA-doped PANI fabric were tested using the DPPH technique. Bars show average readings from three independent experiments. The data were analyzed using one-way ANOVA and Tukey’s hypothesis test. Error bars indicate the standard deviations. Statistical significance was indicated by **** representing *p* < 0.0001.

The results for the DPPH-scavenging activity of bare PES fabric (control), undoped PANI fabric, and *p*TSA-doped PANI fabric (Figure 2 and Table 2) reveal a progressive distinction in the following order: *p*TSA-doped PANI fabric > undoped PANI fabric > bare PES fabric. During the process, one unit of emeraldine salt of PANI could distribute one hydrogen atom and eliminate DPPH free radical. The greater the elimination process of hydroxyl radicals by PANI, the higher its antioxidant activities. Along with its reaction with DPPH, the emeraldine salt of PANI could turn into a pernigraniline state, which has lost its ability to scavenge DPPH. In this case, the dopant in doped PANI fabric donated more hydrogen atoms at approximately 84.83% of its composition, as compared to undoped PANI fabric (35.74%). Therefore, excellent DPPH-scavenging efficiency was clearly seen in protonated PANI fabric.

### 3.3. Antibacterial Performance of PANI Fabrics

**Figure 3:**
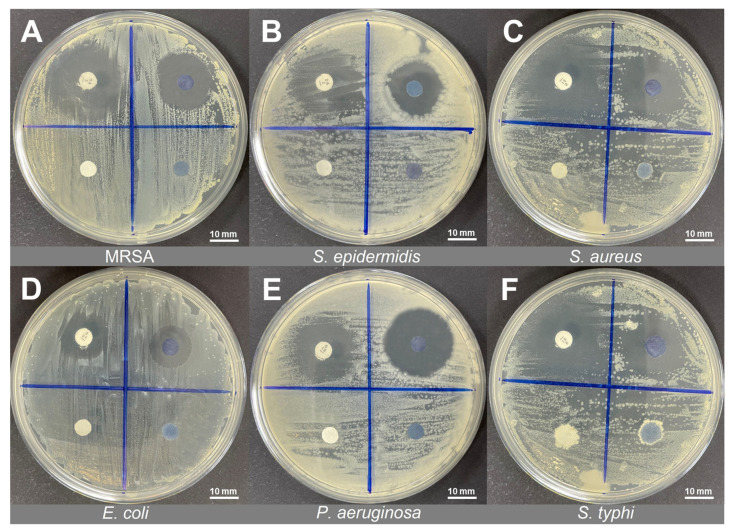
The inhibition zone revealed by bare PES fabric (BF), deprotonated/undoped PANI fabric (UF), protonated/doped PANI fabric (DF), and antibiotic (AB) disc control against (**A**) MRSA, (**B**) *S. epidermidis*, (**C**) *S. aureus*, (**D**) *E. coli*, (**E**) *P. aeruginosa*, and (**F**) *S. typhi*.

The antibacterial action of deprotonated and protonated PANI fabrics was evaluated through the Kirby–Bauer disc diffusion method, which resulted in a substantial indication of antibacterial effects [32]. The inhibition zone (mm) created around the tested fabric on MH agar was measured for antibacterial properties analysis (Figure 3A–F). In this study, Gram-positive bacteria (MRSA, *S. epidermidis*, and *S. aureus*) and Gram-negative bacteria (*E. coli*, *P. aeruginosa*, and *S. typhi*) were chosen to investigate the antibacterial performance of PANI fabrics.

**Figure 4:**
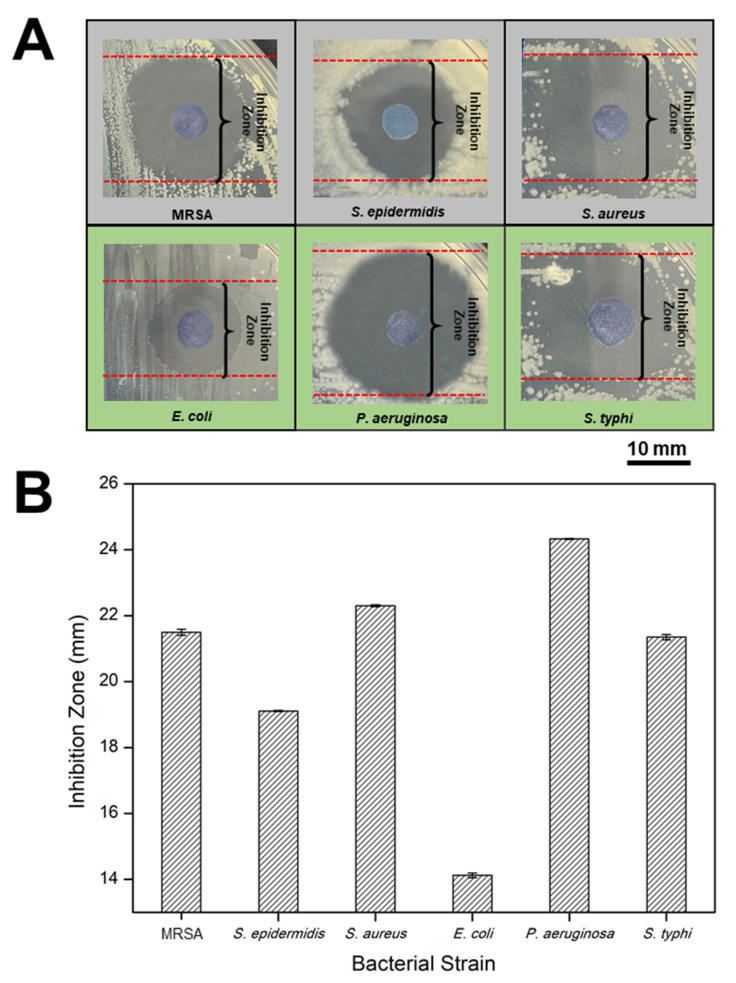
(**A**) Close-up images of inhibition zones formed and (**B**) the inhibition zone values (mm) of each selected bacteria species around the surface of protonated PANI fabric after 18 h of incubation.

The inhibition zone that formed on the bacterial plates around the protonated PANI fabric was observed after 18 h of incubation. Meanwhile, there was no inhibition zone observed on deprotonated PANI fabric or on bare PES fabric. This condition means that the introduction of *p*TSA into PANI-integrated fabrics yielded a noticeable transparent inhibition zone that revealed significant antibacterial properties against the selected bacteria. The inhibition zone diameter of the protonated PANI fabrics was measured an average of three (3) times, and the data are tabulated in Table 3. The close-up images and the inhibition zone of protonated PANI fabric against each strain are shown in Figure 4A,B, respectively.

For the Gram-positive bacteria group, *S. aureus* exhibited a 22.30 ± 0.03 mm breakpoint zone, followed by MRSA at 21.50 ± 0.09 mm, and *S. epidermidis* at 19.11 ± 0.02 mm for the zone diameter breakpoint measurement. In contrast, Gram-negative bacterial groups such as *P. aeruginosa* possessed a 24.33 ± 0.02 mm breakpoint zone, followed by *E. coli* at 14.12 ± 0.07 mm, and *S. typhi* at 21.35 ± 0.08 mm for the zone diameter breakpoint measurement.

Overall, we found that the protonated PANI fabrics showed relatively noticeable antibacterial activity against both Gram-negative and Gram-positive bacteria. In contrast, there were no inhibition zones observed around the deprotonated PANI fabric for both groups of bacteria since no acid was impregnated in the fabric. According to Omar et al. [21], the acid dopant in the fabrics is the critical element involved in establishing the inhibition zones and activity against the bacteria. When the acid dopant leaches out, the fabric is able to inhibit several bacterial isolates on its surface.

**Figure 5:**
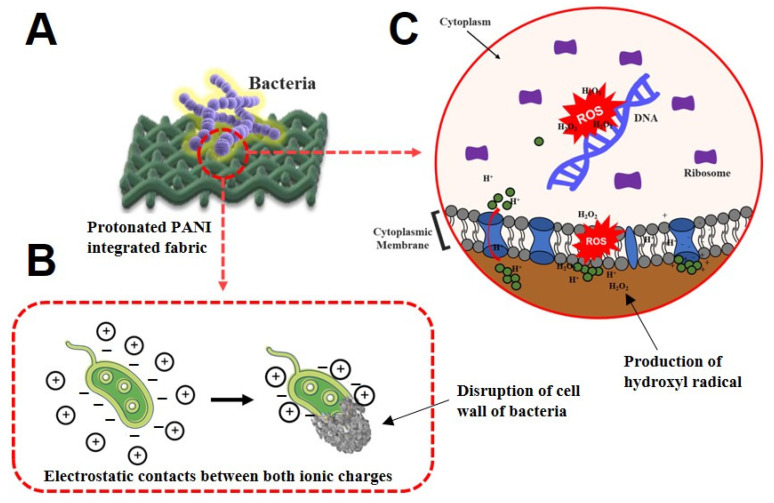
Two possible mechanisms can contribute to the antibacterial activity of protonated PANI fabrics against bacteria. (**A**) The illustration of protonated PANI-integrated fabric when exposed to the bacteria strain. (**B**) Electrostatic contact of cationic compounds in protonated PANI fabric with the anionic charges in the bacterial membrane. (**C**) The disruption mechanism of hydroxyl radicals formed by the production of hydrogen peroxide by PANI reacting with the atmosphere.

There are several tentative mechanisms that can contribute to the antibacterial activity of protonated PANI fabrics against bacteria. When PANI fabric was exposed to the bacteria, cationic antibacterial compounds such as hydrogen (H^+^) were triggered [22]. Consequently, these compounds could penetrate the anionic bacterial membrane. The electrostatic contacts between both ionic charges of protonated PANI and bacterial membranes can interfere with their proliferation, and also result in disruption of their cell walls, which could lead to bacterial cell death (Figure 5A,B).

Our data show that most of the Gram-negative bacteria formed a noticeable inhibition zone around the protonated PANI fabrics compared to those of the Gram-positive bacteria. This can be explained by the difference in cell membrane structures in both groups of bacteria. According to Mahat et al. [10], Gram-negative bacteria have a thinner layer of peptidoglycans compared to Gram-positive bacteria. Due to the exposure of numerous negative charges on their membrane layers, Gram-negative bacteria are attracted to protonated PANI chains. Therefore, PANI chains are tied up to the bacterial membrane and interrupt their natural gradients of ion channels [33]. In contrast, the thicker layer of peptidoglycans of Gram-positive bacteria is very rigid due to their linear polysaccharide chains cross-linked by short peptides. Consequently, this reduces the potential of the PANI chains to penetrate their membrane layers compared to those of Gram-negative bacteria.

Moreover, the existence of hydroxyl radicals formed by the production of hydrogen peroxide by PANI from the fabric to the neighboring atmosphere [34] could aid the bacterial cell membrane disruption mechanism. Referring to Section 3.2, protonated PANI fabric has a huge impact on antioxidant activity at 84.83%. The treated fabric releases reactive oxygen species (ROS) that disrupt multiple biological responses and provokes damage to the bacterial membrane itself (Figure 5C). This study has revealed the potential of protonated PANI fabric in its antibacterial performance, in which *S. aureus* and *P. aeruginosa* showed substantial values of zone diameter breakpoint ranges for antibacterial susceptibility. Therefore, further characterizations that focused on both samples were performed.

### 3.4. Morphological Characterization of Bacteria Strains

**Figure 6:**
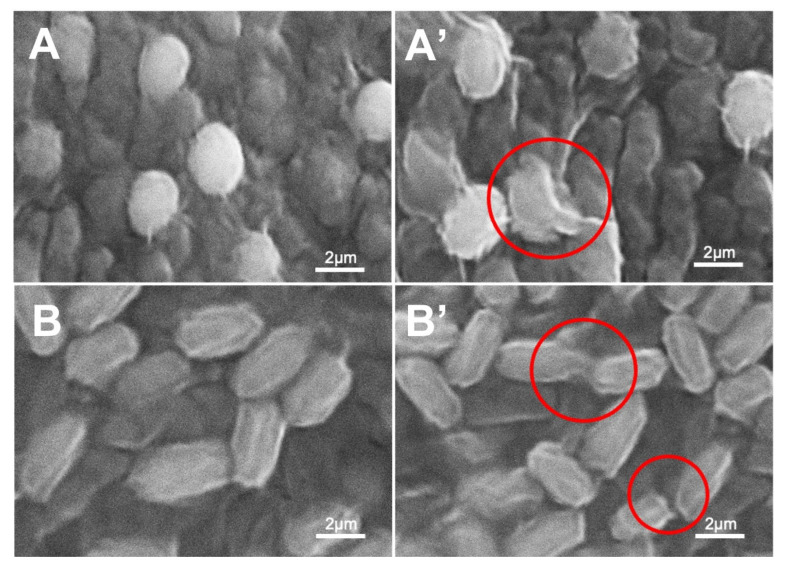
SEM images of (**A**) untreated and (**A’**) treated *S. aureus*; (**B**) untreated and (**B’**) treated *P. aeruginosa*. Treated cells were exposed to protonated PANI fabric. Red circles indicate cell membrane disruption.

SEM was used to elucidate the morphological changes in bacterial cells for *S. aureus* and *P. aeruginosa* strains, before and after exposure to protonated PANI fabric. A smooth surface membrane is visible on the untreated *S. aureus* (Figure 6A) and *P. aeruginosa* cells (Figure 6B). The red circled areas in Figure 6A’,B’ for treated *S. aureus* and *P. aeruginosa*, respectively, show membrane corrugations after treatment with protonated PANI fabric. Surprisingly, wrinkled and damaged cell walls were seen after the exposure to protonated PANI fabric in an 18-hour treatment of both *S. aureus* and *P. aeruginosa*.

As mentioned in Section 3.3, the antibacterial activity of protonated PANI fabric can be explained by the natural reaction of the electrostatic force between the cationic charges of protonated PANI and the anionic charges of the bacterial membrane. This attraction of opposite charges could disrupt the structure of the cells, leading to membrane rupture, as shown in Figure 6A’,B’. The attraction to the negatively charged bacteria’s surface by the cationic amphiphilic antimicrobial of the dopant penetrates the hydrophobic areas of lipid membranes, causing membrane breakdown and membrane damage. According to Rojas et al. [35], the disintegration begins from the outer membrane of the strain, followed by the permeabilization of the inner membrane, and finally, the complete disintegration of both membranes, resulting in the cytoplasmic contents being released. Furthermore, in conjunction with ROS formation in the environment, this mechanism also leads to the leaking of intracellular substances, shrinkage of the cell membrane, and ultimately to cell death [36].

### 3.5. Structural Confirmation and Elemental Identification of Treated PANI Fabric

**Figure 7:**
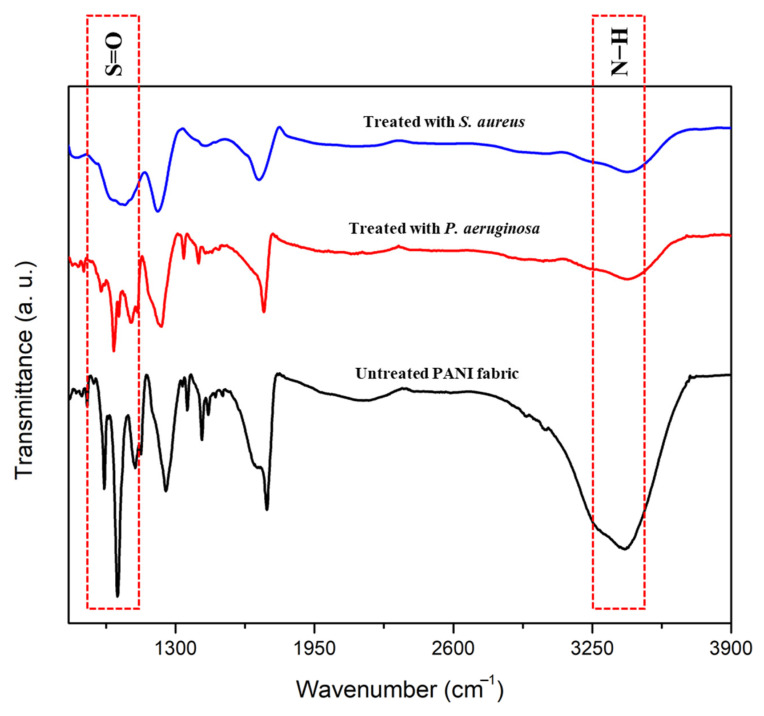
FTIR spectra of untreated PANI fabric, and of PANI fabric treated with *S. aureus* and *P. aeruginosa*.

Figure 7 shows the FTIR spectra, which provide structural confirmation of untreated PANI fabric and treated PANI fabric with bacterial strains. Two (2) significant peaks were confirmed that indicated the presence of PANI and *p*TSA in the fabrics. The general characteristic at ~3073.26 cm^−1^ corresponded to the amine (N–H) stretching peak of PANI [37,38]. Meanwhile, the absorption peak at ~1030 cm^−1^ is assigned to the sulfonate group (S=O stretching vibration) of *p*TSA [39], which is attached to the aromatic ring of protonated PANI. In order to confirm that the antibacterial effects are related to the discharging of dopant, S=O peak intensities in PANI fabrics were observed after 18 h of incubation. Clearly, the S=O band was significantly reduced when the strains were applied. This seems to be related to the changes in protonation and oxidation levels, or to the change in stability of dopant in the conjugated structure of PANI [40] that exhibited the leaching out of dopant from the PANI fabric. Moreover, the decrement of N–H peak intensity was also highlighted after being treated with bacterial strains. Possibly, some of the PANI chains are being expelled together during the leaching out of the dopant from the fabric.

**Figure 8:**
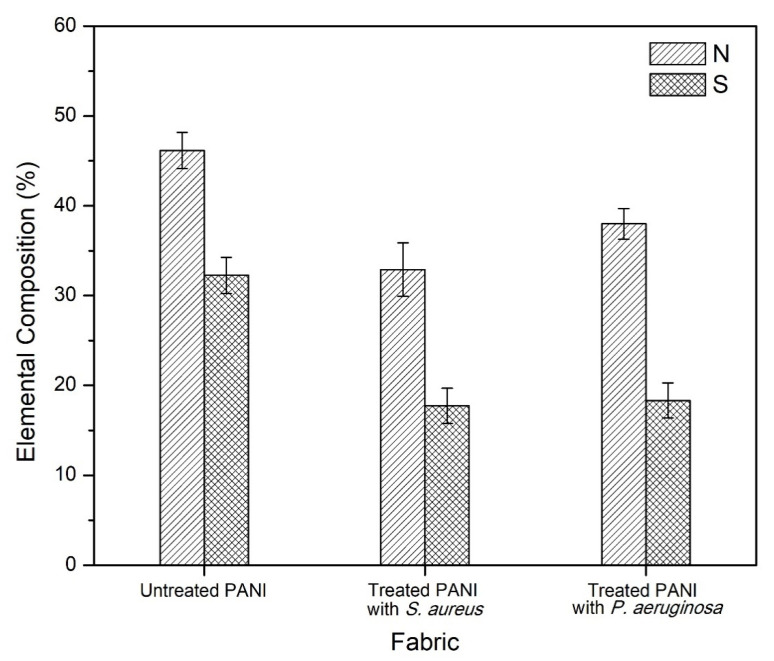
Elemental compositions of untreated PANI fabric, and of PANI fabric treated with *S. aureus* and *P. aeruginosa*.

EDXRF analysis was used in conjunction with FTIR in determining the elemental composition of PANI and *p*TSA in the fabric after the antibacterial assessment. Two main elements were focused on in this study: Nitrogen (N) is attributed to the elemental composition in PANI, whereas sulfur (S) corresponds to the *p*TSA dopant in PANI fabric. These elements displayed some significant changes in composition after being exposed to *S. aureus* and *P. aeruginosa* (Table 4). Based on Figure 8, N and S elements’ composition dropped by 13.29% and 14.97%, respectively, when treated with PANI fabric with the *S. aureus* strain. Meanwhile, 8.18% and 13.94% were exhibited by elements N and S for treated PANI fabric with the *P. aeruginosa* strain, respectively. Compared to these two strains, the disintegration of both N and S was significantly higher in *S. aureus*, which indicates an excellent leaching mechanism for penetrating the bacteria. However, this also contributes to instability of the dopant with the PANI fabric. From another perspective, the weak bonding of dopant with the fabric could affect other potential performances, such as conductivity. Overall, the combination results of EDXRF and FTIR provide essential data to support the possible mechanism (refer to Section 3.3) of discharging the effect of dopant from the fabrics and inhibiting several bacterial isolates when exposed to them.

### 3.6. Conductivity Measurement of Protonated PANI Fabric

**Figure 9:**
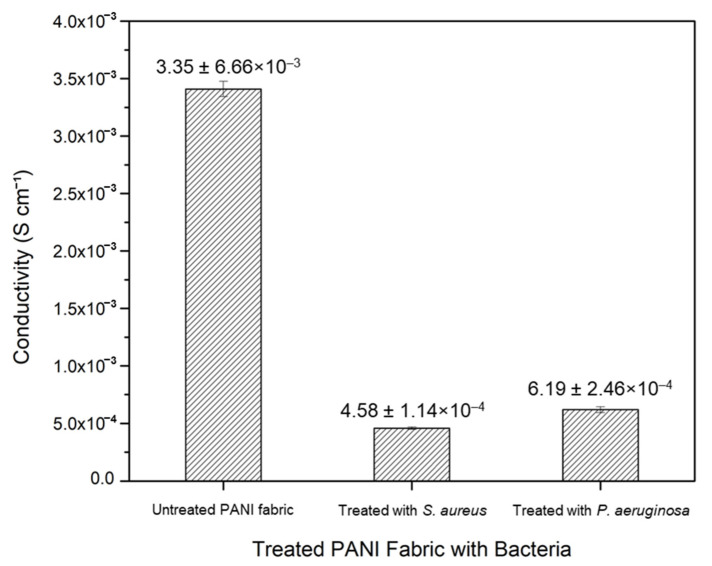
Conductivity values of untreated PANI fabric, and of PANI fabric treated with *S. aureus* and *P. aeruginosa*. Error bars indicate the uncertainties of the reported measurements.

Before the employment of the bacteria, the conductivity value of protonated PANI fabric was recorded at 3.35 ± 6.66 × 10^−3^ S cm^−1^ through the EIS measurement. The incorporation of *p*TSA as a dopant enhances the electrical conductivity of the PANI fabric. This condition permits an electrical charge to pass through the PANI backbone and cause electrical conduction [41]. Here, we report the conductivity value of the fabric upon being treated with both *S. aureus* and *P. aeruginosa* strains, which are recorded and plotted in Table 5 and Figure 9, respectively.

PANI fabrics treated with *S. aureus* and *P. aeruginosa* show significantly reduced conductivity values by one (1) order of magnitude, from 3.35 ± 6.66 × 10^−^^3^ S cm^−^^1^ to 4.58 ± 1.14 × 10^−4^ S cm^−1^ and 6.19 ± 2.46 × 10^−^^4^ S cm^−^^1^, respectively. These drops would significantly disturb the electrical performances of electronic applications; however, the values of both treated fabrics still remain within the range of metallic conductors in the CPs [10,42].

The drop in conductivity values suggest a leaching out of acid that was used to protonate the PANI fabrics. As reported by Mashkour et al. [43], the inhibition zone was created due to the reactivity of the protonated acid in the fabric. Any pathogens on the fabric’s surface were inhibited when the acid leaked. The acid’s positive ions (H^+^) attract the bacteria’s negative ions (Cl^−^), in terms of ionic interaction. Consequently, this attraction of opposite charges can cause damage to the cell wall of the bacteria and hinder electrically dependent functions. The decline in the conductivity value of the protonated PANI fabric may significantly indicate ineffectiveness related to applications for conductive fabrics, but indicates promising impact for its antibacterial properties.

### 3.7. Thermal Stability of PANI Fabric

**Figure 10:**
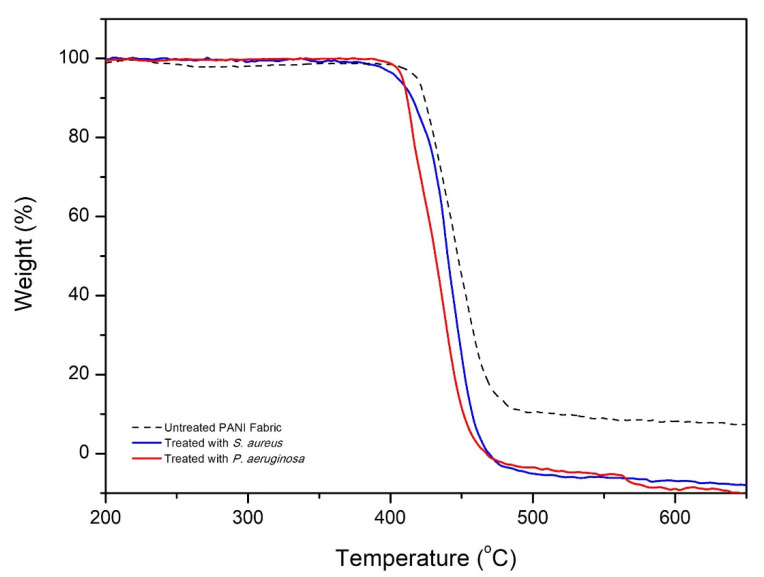
The thermogravimetric weight loss curves of untreated PANI fabric, and of PANI fabric treated with *S. aureus* and *P. aeruginosa* strains.

TGA was used to investigate the thermal stability of the PANI fabric in response to *S. aureus* and *P. aeruginosa*, as shown in Figure 10. It was found that the major thermal decomposition of untreated PANI fabric began at around 413.35 °C, and ended at 503.25 °C with ~90% decomposition. At this phase, the decomposition of the cellulose structure of PES fibers, degradation of PANI moieties, and depletion of dopant are fully complete [16,44]. Additionally, these phenomena could be credited to the interchain cross-linking and breakdown of the PANI backbone from the PES fabric. Based on a recent report [44], dopants such as *p*TSA are likely to significantly improve the thermal stability of untreated PANI fabric. For some reason, these occurrences are due to the structural modification and strong bonding of sulfur with PES fiber.

Focusing on the PANI fabric treated with *S. aureus* and *P. aeruginosa*, the thermal degradation is significantly shifted to 401.07 °C and 398.27 °C at the beginning, respectively. These phases ended at 475.41 °C of total sample decomposition for both samples. It seems that the PANI fabric post-treated with bacteria strains was thermally less stable than untreated PANI fabric (exhibiting larger mass loss), probably as a result of the loss of some dopant composition during antibacterial testing. In this case, the thermal degradation was barely reduced by 2.97% for *S. aureus* and 3.65% for *P. aeruginosa* compared to untreated PANI fabric. The lower amount of sulfur species induced speedy thermal degradation by destroying the radicals generated during the combustion of PES fiber [45]. However, these small changes do not have a big effect on the natural fiber’s properties, and keep virgin fabrics from getting too hot.

## 4. Conclusions

PANI-integrated PES fabrics were developed through an immersion method. A dopant, namely *p*TSA, was used to protonate the fabric and grant antibacterial performance to it. When exposed to the bacteria, some of the dopant leached out of the fabric by electrostatic interaction with bacteria, hence manifesting antibacterial activity. These modified fabrics exhibited remarkably sensitive antibacterial activities against the following Gram-positive bacteria: MRSA, *S. epidermidis*, and *S. aureus*; and against the following Gram-negative bacteria: *P. aeruginosa*, *E. coli*, and *S. typhi*. Among them, *S. aureus* and *P. aeruginosa* yielded promising zone diameter breakpoint range values of 22.30 ± 0.03 mm and 24.33 ± 0.02 mm, respectively. These antibacterial activities were explained by several mechanisms, such as the clashing of electrostatic charge and the production of ROS. The DPPH-scavenging test of protonated PANI fabric expressed high antioxidant activity at 84.83%, compared to that of the bare PES fabric as control. The discharging of dopant was proved by the decreasing intensity of the S=O group and the dropping value of the sulfur element shown in EDXRF analysis. Following this, EIS revealed that the electrical conductivity value of protonated PANI fabric dropped by one (1) order of magnitude, from 3.35 ± 7.81 × 10^−3^ S cm^−1^ to 6.11 ± 7.81 × 10^−4^ S cm^−1^ and 4.63 ± 7.81 × 10^−4^ S cm^−1^ against *P. aeruginosa* and *S. aureus*, respectively. In terms of morphological aspects, SEM analysis showed wrinkled and damaged bacterial cell walls after exposure to protonated PANI fabric. Moreover, TGA proved that the virgin fabric’s thermal stability was maintained. The preparation of protonated PANI fabric suggests promising antibacterial performance due to its inherent dopants such as *p*TSA, and the conjugated backbone of PANI-integrated fabric.

## Figures and Tables

**Table 1:** List of strains and their control antibiotic discs.

Type of Bacteria		Antibiotic Disc (Control)
Gram-positive	MRSA (BAA2094)	Clindamycin (DA—2 µg)
	*S. epidermidis* (ATCC12228)	Clindamycin (DA—2 µg)
	*S. aureus* (ATCC25923)	Clindamycin (DA—2 µg)
Gram-negative	*E. coli* (ATCC1129)	Streptomycin (S—10 µg)
	*P. aeruginosa* (ATCC10145)	Streptomycin (S—10 µg)
	*S. typhi* (ATCC10428)	Streptomycin (S—10 µg)

**Table 2:** Scavenging activity of bare PES fabric (control), undoped PANI fabric, and *p*TSA-doped PANI fabric.

Fabric	Scavenging Activity (%)
Bare PES	NIL
Undoped PANI	35.74 ± 1.01
*p*TSA-doped PANI fabric	84.83 ± 1.50

**Table 3:** Inhibition zone of protonated PANI fabrics in contact with all selected bacteria.

Type of Bacteria		Antibiotic Disc (AB)—Control	Inhibition Zone (mm) of Protonated PANI Fabric with *p*TSA (DF)
**Gram-positive**	MRSA (BAA2094)	Clindamycin (DA—2 µg)	21.50 ± 0.09
	*S. epidermidis* (ATCC12228)	Clindamycin (DA—2 µg)	19.11 ± 0.02
	*S. aureus* (ATCC25923)	Clindamycin (DA—2 µg)	22.30 ± 0.03
**Gram-negative**	*E. coli* (ATCC1129)	Streptomycin (S—10 µg)	14.12 ± 0.07
	*P. aeruginosa* (ATCC10145)	Streptomycin (S—10 µg)	24.33 ± 0.02
	*S. typhi* (ATCC10428)	Streptomycin (S—10 µg)	21.35 ± 0.08

**Table 4:** Elemental compositions of nitrogen (N) and sulfur (S) in untreated PANI fabric, and in PANI fabric treated with *S. aureus* and *P. aeruginosa*.

Fabric	Elemental Composition (%)	
	Nitrogen (N)	Sulfur (S)
Untreated PANI	46.17 ± 2.02	32.25 ± 2.01
Treated with *S. aureus*	32.88 ± 2.98	17.28 ± 1.96
Treated with *P. aeruginosa*	37.99 ± 1.72	18.31 ± 1.95

**Table 5:** Conductivity values of untreated PANI fabric, and PANI fabric treated with *S. aureus* and *P. aeruginosa*.

Fabric	Conductivity (S cm^−1^)
Untreated PANI	3.35 ± 6.66 × 10^−3^
Treated with *S. aureus*	4.58 ± 1.14 × 10^−4^
Treated with *P. aeruginosa*	6.19 ± 2.46 × 10^−4^

## Data Availability

Not applicable.
